# Anti-Biofilm Molecules Targeting Functional Amyloids

**DOI:** 10.3390/antibiotics10070795

**Published:** 2021-06-29

**Authors:** Leticia Matilla-Cuenca, Alejandro Toledo-Arana, Jaione Valle

**Affiliations:** Instituto de Agrobiotecnología (Idab), CSIC-Gobierno de Navarra, 31192 Navarra, Spain; leticia.matilla@unavarra.es (L.M.-C.); a.toledo.arana@csic.es (A.T.-A.)

**Keywords:** biofilm, amyloids, antibiotic resistance, peptides, polyphenols

## Abstract

The choice of an effective therapeutic strategy in the treatment of biofilm-related infections is a significant issue. Amyloids, which have been historically related to human diseases, are now considered to be prevailing structural components of the biofilm matrix in a wide range of bacteria. This assumption creates the potential for an exciting research area, in which functional amyloids are considered to be attractive targets for drug development to dissemble biofilm structures. The present review describes the best-characterized bacterial functional amyloids and focuses on anti-biofilm agents that target intrinsic and facultative amyloids. This study provides a better understanding of the different modes of actions of the anti-amyloid molecules to inhibit biofilm formation. This information can be further exploited to improve the therapeutic strategies to combat biofilm-related infections.

## 1. Introduction

### 1.1. Biofilm Related Infections

Nosocomial infections related to the use of medical devices are associated with a high risk of mortality and increased economic costs [1]. These infections are mainly caused by opportunistic bacteria such as *Enterococcus faecium*, *Staphylococcus aureus*, *Klebsiella pneumoniae*, *Acinetobacter baumannii*, *Pseudomonas aeruginosa*, and *Enterobacter* species from the ESKAPE group, many of which are resistant to commonly used antibiotics [2]. The rapid appearance of antibiotic resistance in these bacteria is associated with a global increase in antibiotic consumption in the fields of health care, agriculture, and in the environment, in addition to their inappropriate utilization related to inadequate dosing or antibiotic selection [3,4,5]. The difficulty of treating healthcare-associated infections is also intensified by the common trait of the opportunistic bacteria to form biofilms. Bacteria are able to form multicellular communities on the surface of biomaterials, mucosa, tissues, and secretions, and express a self-generated extracellular polymeric matrix in response to myriad signals. The extracellular matrix, composed of polysaccharides, surface proteins, nucleic acids, and amyloid fibers, protects the microbes from external insults and adverse environmental conditions. The biofilm matrix acts as a physical and chemical barrier that reduces the rate of antimicrobial penetration. In addition, it can generate microenvironments that antagonize the action of antibiotics and promote the ability of bacteria to generate persister cells with increased drug tolerance [6,7,8,9]. Thus, bacteria in biofilms are less sensitive to antibiotics than the same strain growing in suspension, resulting in a 10- to 100-fold decrease in their susceptibility [10]. Because conventional antibiotics fail to successfully treat biofilm-associated infections, novel therapeutic solutions are in urgently needed. Efforts are currently underway to develop new therapeutic approaches that focus on preventing the synthesis or the assembly of the biofilm matrix components to render bacteria sensitive to antibiotic treatments.

### 1.2. Amyloids as Structural Scaffolds of the Biofilm Matrix

Amyloids are traditionally associated with several incurable degenerative human diseases. However, numerous studies have indicated that microorganisms make use of amyloidogenic proteins for a number of non-pathological processes [11]. In recent decades, amyloids have been considered to be essential components of the biofilm, providing consistency and viscoelasticity to the extracellular biofilm matrix [12,13,14,15]. Amyloids are highly ordered fibrillar proteins with a β-sheet secondary structure and a strongly conserved quaternary cross-β structure [16]. To a lesser extent, α-helices can replace the β-strands, thus forming cross-α fibrils [17,18]. The amyloid structure binds a range of specific dyes such as Congo red, Thioflavin T (ThT), and ProteoStat, which, combined with other biophysical techniques, such as solid-state NMR and Fourier-transform infrared spectroscopy (FTIR), are normally used to determine the amyloidogenic features of a protein [19,20,21,22]. Due to the well-ordered structure of β-strands that are aligned perpendicular to a fibril axis, amyloids are an effective extracellular building material. They are mostly resistant to harsh denaturing conditions and proteolytic cleavage. In addition, polymerization of amyloidogenic proteins occurs through a nucleation-dependent self-assembly process, in which starter amyloid aggregates provide a conformational scaffold that facilitates the assembly of polymeric subunits into the amyloid state in the absence of energy. Due to this seeding mechanism, the amyloid conformation is suitable under conditions in which the energy is limited [12,23]. 

Taking into account that amyloids are widely distributed components of the biofilm matrix in many bacteria, they can be considered excellent targets for anti-amyloid drugs to reduce biofilm formation. This review aims to summarize the current knowledge of the bacterial amyloids and the different mechanisms of action of the anti-amyloid molecules to inhibit biofilm formation. 

## 2. Functional Amyloids of the Biofilm Matrix

In recent years, an increasing number of amyloid components of the biofilm matrix have been identified, purified, and closely investigated [15]. Based on the structural and functional state of the amyloidogenic proteins, it is becoming popular to group them into intrinsic and facultative amyloids (Table 1) [24].

### 2.1. Intrinsic Amyloids

The polymeric structure of intrinsic amyloids is the result of a dedicated system that controls the secretion and assembly of the monomeric building blocks. Indeed, the amyloidogenic subunits do not have a folded state prior to forming the amyloid fibers. The amyloid conformation represents the primary structural and functional state of the protein. Examples of amyloids from this group are described below.

#### 2.1.1. Curli (*csgBAC-csgDEFG* Genes)

**Figure 1:**
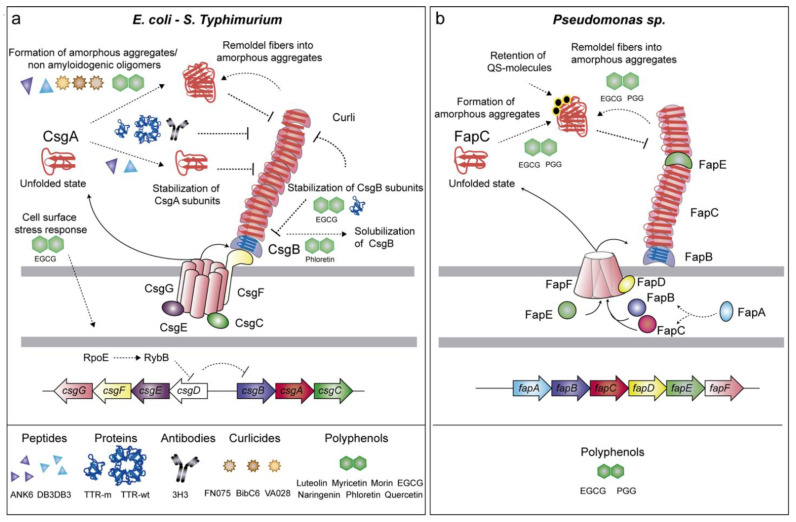
Schematic representation of the genes involved in the formation of intrinsic amyloids, the drugs targeting such structures and their mechanism of action. (**a**) The formation of *E. coli* and *S.* Typhimurium curli amyloids is affected by several drugs, which act by different mechanisms: (i) stabilization of CsgA monomeric subunits; (ii) formation of amorphous or non-amyloidogenic aggregates; (iii) disaggregation of already-formed fibers; (iv) prevention of CsgB polymerization; (v) solubilization of CsgB; (vi) activation of the cell surface stress response, which reduces the expression of the curli regulator CsgD. (**b**) *Pseudomonas* Fap amyloids are inhibited by EGCG and PGG polyphenols, which lead FapC to off-pathway oligomers, remodeling fibers into amorphous aggregates and retaining quorum-sensing molecules.

The curli fimbriae present in *E. coli*, *Salmonella,* and other Enterobacteria is one of the most well-characterized functional amyloid systems. The curli-specific genes are disposed in two divergent operons *csgBAC* and *csgDEFG* [25]. CsgA is the major curli subunit that structurally forms the amyloid fibers. To achieve this, the minor curli subunit CsgB acts as a nucleator of CsgA assembly (Figure 1a). To avoid the formation of toxic oligomers inside the cell, the periplasmic protein CsgC acts as a chaperone that maintains CsgA as a soluble and unstructured monomer, thus preventing the amyloid state [26]. The *csgDEFG* operon code for proteins that are involved in the regulation, stabilization, and secretion of CsgA and CsgB subunits [26,27,28]. CsgA and CsgB reach the extracellular milieu, due to a pore-like structure formed by CsgG in the outer membrane [29]. The accessory proteins CsgF and CsgE are associated with CsgG and contribute to the proper assembly of curli fibers [30]. In addition, CsgF interacts with CsgB, ensuring the attachment of the subunit on the cell surface and thus, the fiber formation [30]. CsgD controls at the transcriptional level the expression of the *csgBAC* operon [31,32] which is triggered by environmental and chemical signals such as osmolality, oxygen, and temperature [33,34]. Curli are implicated in surface colonization, cell–cell contact, biofilm matrix scaffolding, protection against antimicrobial agents and desiccation. Moreover, curli play an important role in the interaction with host cell receptors and the immune system [35,36,37,38,39]. 

#### 2.1.2. Fap (*fapA-F* Genes) 

Fap fibers are produced by *Pseudomonas* spp. as part of the biofilm matrix [52]. The Fap fiber machinery is encoded in the operon *fapA-F*. The Fap fibers are assembled by the major structural subunit FapC, and the two minor subunits FapB and FapE. FapB is required for fiber polymerization whereas FapE acts as an extracellular chaperon-like protein (Figure 1b). The three proteins are exported outside the cell through the pore-like structure formed by FapF [53]. FapA works as a chaperone of the fiber monomers during the secretion process whereas FapD, a cysteine protease located in the periplasm, is required for FapC externalization [53]. The assembly of Fap amyloids in the biofilm enhances cell aggregation and attachment conferring protection against chemical and mechanical attack [52]. In addition, Fap amyloids increase the overall hydrophobicity and stiffness of the biofilm, thus preventing bacteria desiccation [54]. 

#### 2.1.3. Chaplins and Rodlins 

Filamentous bacteria, such as *Streptomyces,* grow above humid surfaces forming branching hydrophobic structures called aerial hyphae that ultimately result in hydrophobic spores [55]. The amyloidogenic proteins, chaplins and rodlins are involved in the formation of these [43,44]. There are eight chaplins (ChpA–H) and two rodlins (RdlA and RdlB). The long chaplins (ChpA–C) have a C-terminal sorting signal for attachment to the peptidoglycan via a sortase-mediated covalent bound with the LAXTG motif and two chaplin domains that are highly hydrophobic, whereas the short chaplins (ChpD-H) have one chaplin domain [56]. To date, because long chaplins cannot be extracted from the cell walls of aerial hyphae due to the covalent linkage, only short chaplins have been shown to form β-sheet structures and thus amyloid-like fibers [57]. It is therefore possible that ChpA-C serve as anchoring sites for ChpD-H chaplins to form amyloid fibers, and that ChpE is responsible for the coordination of the polymerization of the fiber subunits in a high pH-dependent mechanism [58]. In addition to chaplins, the RodA and RodB rodlins polymerize into the hydrophobic structure known as the rodlet layer, which covers the surface of aerial hyphae and spores. Amyloid chaplins are aligned into ordered rodlets by the action RdlA and RdlB, only the latter is able to form amyloid fibrils in vitro [59]. 

### 2.2. Facultative Amyloids

The facultative amyloids include proteins with a dual function. They are secreted in a functional globular folded state that, under certain conditions, changes their conformation to an amyloid fold [12,24]. Two types of facultative amyloids can be differentiated. One type comprises secreted monomeric proteins, such as *S. aureus* phenol-soluble modulins (PSMs) and *Bacillus subtilis* TasA, which assemble into amyloid fibers upon conformational change occurs. The other group includes protein domains with amyloidogenic features, which has been previously processed from a native surface protein (e.g., *S. aureus* Bap, *Enterococcus faecalis* Esp, and *Streptococcus mutans* P1, WapA, and SMU_63C). In their native conformation, the facultative amyloids of the former group can act as antimicrobials and toxins, whereas the latter work as cell surface adhesins. Both turn into matrix scaffolds when they polymerize into amyloid structures.

#### 2.2.1. Phenol-Soluble Modulins (PSMs) 

**Figure 2:**
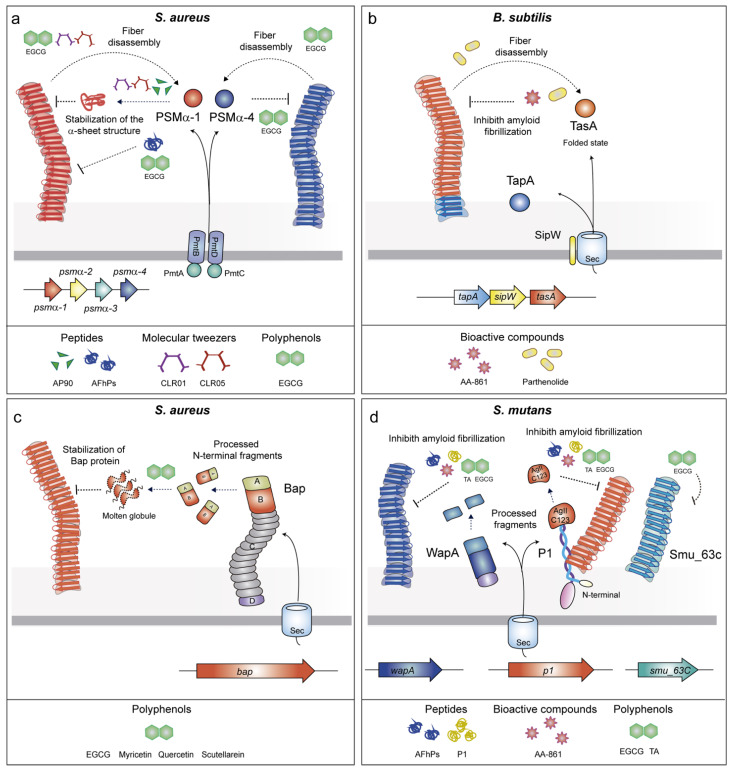
Schematic representation of the genes involved in the assembly of facultative amyloids, the drugs targeting such structures and their mechanism of action. (**a**) Drugs targeting *S. aureus* PSMs act by several mechanisms that involve the stabilization of PSM α-sheet structure and fiber disassembly. (**b**) *B. subtilis* TapA polymerization is inhibited by AA-861 and parthenolide. (**c**) The flavonoids quercetin, myricetin, and scutellarein inhibit polymerization of the Bap amyloid aggregates by stabilization of the Bap protein. (**d**) Polymerization of the amyloids *S. mutants* WapA and AgII/C123 is inhibited by AA-861, Tannic acid (TA), and EGCG and indirectly by the AFhPs and P1 peptides. SMU_63c amyloids are inhibited by EGCG.

Phenol-soluble modulins (PSMs) are small alpha-helical amphipathic peptides involved in the virulence of *S. aureus* [60,61]. *S. aureus* expresses four α-PSMs (PSMα1–PSMα4) encoded in the *α-psm* operon, two β-PSMs (PSMβ1 and PSMβ2) encoded in the *β-psm* operon and γ-PSM (δ-toxin) produced from RNAIII, a regulatory RNA of the Agr system [62,63,64]. PSMs lack the typical Sec-signal and their secretion is carried out by an ATP-binding cassette (ABC) transporter [65]. Once at the extracellular media, PSMs may either work as soluble proteins having antimicrobial and immunomodulating activities [66,67,68,69] or change the conformation and polymerize into functional amyloids (Figure 2a). PSMα1 and PSMα4 form aggregates showing a typical amyloid cross-β structure [45] whereas PSMα3 aggregates display an unusual cross-α structure [17]. This structural polymorphism reveals different bacterial functions. Although PSMα1 and PSMα4 participate in biofilm formation, PSMα3 is involved in cytotoxicity of human T cells, probably due to its particular amyloid architecture [17]. 

#### 2.2.2. TasA

The *B. subtilis* biofilm contains functional fibers with amyloidogenic properties. The main amyloid component is TasA (translocation-dependent antimicrobial spore component), which self-assembles into amyloid fibers (Figure 2b) [46]. The accessory protein TapA (TasA anchoring and assembly protein) assists in the formation of TasA fibrils onto the cell surface [47]. SipW is a membrane-bound peptidase that cleaves the TapA and TasA signal peptides before secretion [70]. Structural analyses have shown that TasA is secreted in a globular state that transits to an amyloid conformation under environmental conditions such as acidic pH and hydrophobicity [71]. In addition to its structural role in the multicellular behavior, TasA maintains the cell membrane stability and prevents excess cell death under biofilm growth conditions [72].

#### 2.2.3. Biofilm Associated Proteins (BAPs)

BAPs are high molecular weight proteins present on bacterial cell surfaces and are characterized by a multi-domain architecture [73,74]. The paradigm *S. aureus* Bap protein is organized in several domains (Figure 2c) including a putative Sec-dependent signal peptide for their export followed by a N-terminal domain, which comprises region A and B, the latter carries the EF-hand motifs for calcium binding [75,76]. Thereupon, a core domain formed by tandem repeats (C region) and a region D at the C-terminus, which contains the LPXTG motif for anchoring the protein to the cell wall. Bap forms amyloid fibers under specific environmental circumstances [48]. Once the protein is covalently anchored to the peptidoglycan, the N-terminal region is processed and released to the extracellular media. As a result, fragments of Bap adopt an unstable molten globule-like state. When acidic pH and low concentration of Ca^2+^ are found in the medium, the molten globule switches to an aggregation-prone conformation that enables self-assembly into amyloid fibers that promote biofilm formation (Figure 2c) [48]. In contrast, when Ca^2+^ is available, it binds to the EF-hand motifs, stabilizing Bap in its native folded state and impairing the polymerization of the amyloid fibers and biofilm formation [48,76]. It is notable that Bap carries out additional functions in its primary globular state. It acts as an adhesin, promoting the primary attachment of *S. aureus* to abiotic surfaces and thus playing an important role in initial stages of biofilm formation [75]. In addition, Bap is involved in the pathogenesis of *S. aureus* by improving the adhesion to epithelial cells of mammary glands [77] but exerting a protective role by preventing *S. aureus* entry into epithelial cell through binding to the Gp96 cell host receptor [78].

The Enterococcal surface protein (Esp) is a Bap-orthologous protein present in *E. faecalis* [79,80]. The multi-domain configuration of Esp is similar to that of Bap. The Esp N-terminal domain and the region C share 26% and 23% of identity with the corresponding Bap domains, respectively [49]. Analogously, the Esp N-terminal domain is sufficient to induce biofilm formation [81]. Our group has recently described the capacity of the Esp N-terminal region to form amyloid-like fibers in a pH-dependent manner [49]. The similarities observed between Bap and Esp suggest that the dual function of amyloid/adhesin may be widespread among members of the BAP family. 

#### 2.2.4. P1 Adhesin/WapA/SMU_63c 

Another example of a surface-associated adhesin with amyloidogenic properties is the P1 protein (also known as AgI/II, PAc, SpaP or antigen B) of *S. mutans.* This P1 is a large protein that is covalently anchored to the cell wall through its C-terminal LPXTG motif. P1 is able to bind extracellular proteins [82,83,84]. The tertiary structure is a prolonged stalk with a globular domain that contains β-sheets. P1 can be processed and its C-terminal domain (AgII or C123 fragment) can be loosely associated with the covalently anchored protein (Figure 2d) [85]. The C123/AgII fragment contains the amyloid-forming moiety that may be implicated in the formation of fibrillar structures with amyloid properties [50,86]. In addition, the wall-associated protein A (WapA) and the secreted protein SMU_63c of *S. mutans* contribute to the fibrillar structures depending on the pH [51]. As the P1 adhesin, WapA is processed and forms amyloid fibers at neutral pH, whereas SMU_63c does not need to be processed and forms amyloid structures at acidic pH. Overall, this evidence suggests that environmental conditions are crucial for the regulation of *S. mutans* amyloid fibers.

## 3. Drugs Targeting Amyloid-Structured Biofilms

Amyloid fibers appear to be common biofilm matrix elements in many pathogenic bacteria. Several approaches have been focused on searching for compounds that either alter the expression of the elements involved in the amyloid production or interrupt their assembly. The identification of the anti-amyloid compounds to disassemble biofilms has been carried out by means of high-throughput screening of active molecule collections, the design of peptides based on the structural biology of the amyloidogenic segments or the repurposing of molecules designed against human amyloids. The most representative drugs active against amyloid-related biofilms and their mechanisms of action are discussed below and shown in Table 2.

### 3.1. Anti-Amyloid Peptides

The design of peptides binding to the domains involved in amyloid fibrillation is one of the most promising strategies used to interrupt amyloid maturation and consequently biofilm formation. Based on the structural similarities existing between the amyloidogenic domains derived from CsgA and those from human amyloids, Perov and collaborators found that the D-enantiomeric peptides (ANK6 and DB3DB3) inhibited the fibrillation of CsgA (Table 2) [87]. ANK6 and DB3DB3 peptides were optimized for their potential to remove amyloid-β (Aβ) oligomers associated with Alzheimer’s disease. The D-peptides bound to CsgA, thus promoting the stabilization of the monomer subunits or inducing the formation of amorphous aggregates, which were unable to assemble into amyloid fibers (Figure 1a). The peptides inhibit the fibrillation of the CsgA spine segments contained at the R1 and R5 repeats. The inhibitory action of the peptides appears to be specific to the secondary structure of the protein because ANK6 and DB3DB3 do not have any effect on cross-α amyloid-like fibrils formed by PSMα3 fibrillation [87]. This distinction was further exemplified in a pioneering study that designed peptides that were complementary to the α-sheet of PSMα1. It is often considered that, because amyloids contain a cross-β-structure, the intermediate aggregative species must also contain a β-structure. However, this is not the case for several amyloids, such as PSMα1, which undergo a transition from α-helix→α-sheet→β-sheet during aggregation [88]. The anti-α-sheet peptide AP90 targets the transitional α-sheet structure of PSMα1, leading to a reduction of amyloid fibrils in vitro (Figure 2a). Consequently, AP90 effectively reduces biofilm formation by *S. aureus* through the interaction with PSMs [88].

Another interesting strategy for treating microbial infections is based on the use of amyloidogenic peptides derived from bacterial amyloids [100]. Synthetic aggregation-prone peptides from *Staphylococcus epidermidis* (C30, C29, Hit1, Hit50) penetrate bacteria, thus causing toxic protein aggregation of polypeptides in the cytosol, which leads to bacterial death [101]. Interestingly, aggregating peptides are toxic to bacteria, but not to human cells. However, further investigations are needed to test whether these peptides may have an effect on protein aggregation and toxicity in human cells that express amyloids such as α-synuclein or Aβ.

Furthermore, short peptides derived from the amyloidogenic fragment C123 of the adhesin P1 of *S. mutans* display strong anti-biofilm properties with small or no cytotoxic effect [89]. Simulated amyloid forming peptides predicted from C123 have been synthesized (AFhPs). AFhPs inhibit biofilm formation in a wide range of microorganisms including Gram-positive and Gram-negative bacteria and fungi. These peptides aggregate into rigid amyloid fibers that agglutinate microbial cells into clusters [89], thus leading to the formation of aggregates that impair the establishment of new biofilms. It was proposed that the carbohydrates of the microbial cell wall would be the binding targets of AFhPs rather than the surface-exposed amyloids. Moreover, lipid membranes may accelerate AFhP fibrillation. However, the role of these structures in the anti-biofilm activity of AFhPs remains to be elucidated. 

Similarly, an anti-biofilm activity was identified for the P1 peptide, a synthetic peptide of 24 amino acids (different from the P1 protein of *S. mutans*), which was designed from the repeated motifs of the Ixodes tick antifreeze glycoprotein (IAFGP) sequence [102]. The P1 peptide is able to inhibit biofilm formation of *S. mutants* and other Gram-positive bacteria but does not possess bactericidal activity [103]. The anti-biofilm effect is associated with P1 aggregation and the formation of antiparallel β-sheets. However, further experiments are needed to determine whether the anti-biofilm effect is caused by the intermolecular interactions of P1 aggregates with bacterial amyloidogenic proteins [103]. 

### 3.2. Anti-Amyloid Proteins

The human amyloid precursor transthyretin (TTR) is a 55 kDa homotetramer protein mainly produced by the liver. Interestingly, TTR is structurally similar to the curli inhibitor CsgC, and both share a stable folded β-sheet-rich structure [104,105]. TTR is involved in the transport of thyroxine (T4) and retinol in plasma and cerebrospinal fluid. Misfolded protein monomers, derived from the tetrameric protein TTR, can be accumulated as fibrillar deposits in different organs and cause the most common form of hereditary transthyretin amyloidosis. By comparison, the homotetrameric form of the protein has the ability to inhibit amyloid formation of amyloidogenic proteins, such as Aβ, the N-terminal domain of the bacterial hydrogenase HypF and the human islet amyloid polypeptide [106,107,108]. Taking into account the anti-amyloid properties of TTR, in a remarkable work, Jain et al. examined the effect of TTR on CsgA amyloid assembly [109]. The incubation of CsgA with the tetramer precursor (Wt-TTR) retarded CsgA polymerization, whereas the incubation with the monomeric form (M-TTR) strongly prevented the formation of CsgA protofibrillar and fibrillar structures (Figure 1a). Interestingly, M-TTR also inhibited the assembly of CsgB into amyloid aggregates. Therefore, both Wt-TTR and M-TTR inhibited wrinkled pellicle biofilms formed by *E. coli*; however, the inhibitory effect of M-TTR was significantly more potent than that produced by Wt-TTR. It is possible that Wt-TTR requires tetramer dissociation to liberate the monomers that are considered the active forms for fibril inhibition. However, the use of a highly stable TTR tetramer form (TTR T119M) inhibited CsgA fibrillogenesis, indicating that the Wt-TTR tetramer dissociation was not required for such inhibition [109].

### 3.3. Antibodies as Native-State Stabilizing Agents

The administration of antibodies or engineered affibodies has been proposed as one of the most promising therapies to reduce amyloid polymerization [110]. Antibody-based immunotherapy has been used to reduce the amyloid amount by stimulating the clearance of the misfolded aggregates by the immune system cells [111]. In addition, antibodies can be used as native-state stabilizing agents that bind to the aggregation-prone proteins, increasing their stability and impairing the aggregation tendency of human amyloidogenic proteins [112,113]. A recent pioneering study proposed the use of antibodies as native-state stabilizing agents to reduce bacterial amyloid aggregation [90]. The human monoclonal antibody mAB 3H3, which specifically binds Aβ and other human amyloids, such as immunoglobulin light chain, transthyretin, and tau, also binds the curli amyloids. Antibody mAB 3H3 inhibits the initiation of CsgA polymerization and therefore, curli fiber elongation (Figure 1a). The incubation of *S.* Typhimurium with mAB H3 alters the structure of the biofilm. Consequently, due to changes in the biofilm architecture, antibiotic treatment and macrophage uptake are more effective at removing *S.* Typhimurium biofilms in vivo from an implanted catheter mouse model. Because mAB 3H3 binds and inhibits polymerization of human and bacterial amyloids with significant differences in the primary amino acid sequence, it has been proposed that mAB H3 should bind to an intermediate conformation that appears to be common in different amyloidogenic proteins [90].

### 3.4. Molecular Tweezers 

Molecular tweezers are supramolecular host molecules comprised of two aromatic arms linked by a spacer that has the capacity to bind guest molecules with high specificity. Therefore, these macromolecules may work as singular molecular receptors for specific targets that can be used in numerous applications in biology and medicine [114]. Among the different applications, it was reported that a lysine-binding tweezer (CLR01) inhibits the self-assembly of different human amyloidogenic proteins, including Aβ protein, tau, and α-synuclein in vitro and in vivo [115,116]. CLR01 alters the assembly process and promotes the formation of non-amyloidogenic structures that can be efficiently removed by host clearance mechanisms (Figure 2a). In addition, CLR01, and its derivative CLR05, reduced biofilm formation of *S. aureus* [91]. CLR01 and CLR05 inhibit PSMα1 fibrillization and disintegration of mature fibrils. In particular, CLR01 interferes with the secondary structure of PSMα1and inhibits their polymerization, possibly through the enclosure of lysine residues of PSMα1. CLR05, which exhibits less affinity for the lysine residues, inhibits *S. aureus* biofilm formation more strongly than CLR01. The “truncated” molecular tweezer CLR3, which was used as a negative control, did not affect *S. aureus* biofilms and had a small impact on PSMα1 assembly. Although these molecules could be promising drugs to treat staphylococcal infections, further characterization of the interactions between CLR01 and CLR05 with PSMα1 is necessary to determine their potential as anti-biofilm molecules in vivo.

### 3.5. Anti-Amyloids Based on Pilicides and Curlicides 

Pilicides have been extensively used as anti-virulence compounds that block the formation of pili in *E. coli* [117]. Derivatives of the dihydrothiazolo ring-fused 2-pyridone pilicide 1, such as FN075, BibC6, and VA028, gain anti-amyloid properties and inhibit Aβ polymerization in vitro [118]. Thus, FN075, BibC6, and VA028 were tested for their ability to inhibit curli assembly. The compounds inhibited curli biogenesis and CsgA polymerization in vitro [92]. The mechanism by which FN075 inhibits CsgA fibrillization involves the formation of non-amyloidogenic oligomers (Figure 1a) [119]. FN075 and BibC6, at least, prevent amyloid-dependent biofilm formation at the air–liquid interface and all of the compounds inhibit biofilm formation by the uropathogenic *E. coli* strain UTI89 on PVC microtiter plates [92]. Moreover, FN075 was effective at inhibiting *E. coli* UTI89 in vivo in a murine model of urinary tract infection [92]. Therefore, a new class of biofilm inhibitors, termed curlicides, was stablished with the capacity to block curli. The double curlicide–pilicide property of FN075 provides additional therapeutic value because it could inhibit the formation of several pili and fibers that are important for virulence and biofilm formation.

### 3.6. Bioactive Compounds with Anti-Amyloid Properties

Using throughput screening for molecules with anti-biofilm activity, Romero and collaborators found two active molecules, a benzoquinone derivative (AA-861) and a sesquiterpene lactone (parthenolide) with the capacity to alter the wrinkly biofilm phenotype of *B. subtilis* [93]. The AA-861 and parthenolide did not affect *tasA* gene expression but they inhibited the polymerization of TasA into amyloid fibers (Figure 2b). AA-861 was more efficient at hindering TasA polymerization, whereas parthenolide was more effective at disrupting already-formed biofilms. These results indicated a synergistic activity of both molecules to inhibit biofilm formation. They also showed that AA-861 and parthenolide were effective at inhibiting biofilm formation of *E. coli* and *B. cereus* but did not showed any detectable activity against *S. aureus* and *P. aeruginosa* biofilms. However, it was not clarified whether their anti-biofilm effect was via direct inhibition of the CsgA and TasA polymerization, respectively, or due to an alternative mechanism. In a latter work, it was shown that AA-861 and not parthenolide was also effective at inhibiting *S. mutans* biofilm formation [51]. AA-861 inhibited the fibrillization of the amyloidogenic moieties of the adhesin P1 (C123 and antigen A) and WapA in vitro, whereas SMU_63c was completely unaffected by this molecule (Figure 2d). These results validate the broad-spectrum activity of AA-861 on different amyloids.

### 3.7. Polyphenols 

Polyphenols are naturally occurring secondary metabolites, and are widely spread in fruits, vegetables, seeds and plant-derived oils. They are considered to be powerful anti-amyloidogenic compounds due to their physicochemical features. Polyphenols can affect the fibrillation of amyloidogenic proteins involved in neurodegenerative diseases by inhibiting nucleation and elongation or redirecting to “off-pathway” aggregation [120,121,122]. The structural similarities between human and bacterial amyloids suggest that polyphenols may disturb the biofilm matrix by interfering with the bacterial amyloids. 

A potent anti-amyloidogenic polyphenol is epigallocatechin gallate (EGCG), which is the major active polyphenol in green tea (*Camellia sinensis*). EGCG has an indiscriminate protein binding and inhibits the function of a variety of proteins. Its effect on the oligomerization of multiple human amyloids such as Aβ [123,124,125], α-synuclein [124,126,127,128], islet amyloid polypeptide [129,130], huntingtin [131], and tau protein [132] has been extensively demonstrated. In these proteins, EGCG induces the formation of partially stable oligomers, which are unable to form amyloid fibers [125]. EGCG can also reorganize preformed amyloid fibrils into amorphous aggregates [133]. The anti-amyloidogenic properties of EGCG have been also extended to bacterial amyloids. Serra and collaborators showed that EGCG interfered with the assembly of curli amyloid subunits, and found that EGCG prevented CsgB from adopting an amyloid conformation and prevented CsgA from polymerization into amyloid fibers (Figure 1a) [94]. In addition to its direct effect on curli polymerization, EGCG triggers the cell surface stress response governed by the alternative sigma factor RpoE, which finally induces the downregulation of the biofilm regulator CsgD [94]. 

At the same time, it was shown that the EGCG acts against Fap amyloid fibers in *P. aeruginosa* [96]. EGCG both remodels already-formed Fap fibrils into amorphous aggregates and inhibits FapC fibrillation by redirecting FapC to off-pathway oligomers (Figure 1b) [96]. EGCG can inhibit FapC fibril formation, even in the presence of bacterial amphiphiles, such as rhamnolipid and LPS, otherwise they promote fibril formation [134]. In addition to EGCG, the gallotannin penta-O-galloyl-d-glucose (PGG) was found to be a strong inhibitor of Fap fibrillization [134]. PGG induced the formation of oligomers and larger non-fibrillar aggregates, that had low β-sheet content and that were remarkably stable. EGCG and PGG reduce the amyloid content in *P. aeruginosa* biofilms, leading to a higher antibiotic susceptibility of bacterial cells [95,96]. 

An alternative mechanism of biofilm inhibition by EGCG involved the bacterial quorum-sensing system [96]. It was speculated that the FapC amorphous aggregates formed by EGCG increased the binding and retention of the quorum sensing molecule pyocyanin, thereby reducing the autoinducer concentration [96]. Therefore, the administration of the polyphenols EGCG and PGG was proposed as a promising treatment for cystic fibrosis patients in which Fap fibers play an important role during *P. aeruginosa* biofilm-mediated infections. 

The anti-amyloid effect of EGCG was also extended to other biofilm-associated amyloids including *S. mutans* P1 as the main target and WapA and SMU_63c as secondary targets and *S. aureus* PSMα1 and PSMα4 (Figure 2a,d) [51,97]. Due to the extensive anti-amyloid properties of EGCG, in vivo studies will be needed to determine its potential as a therapeutic compound against biofilm-related infections.

Polyphenols of the flavonoid group, such as luteolin, myricetin, morin, quercetin, phloretin and, to a lesser degree, naringenin, also inhibited the assembly of the curli amyloid fibers. Most of these flavonoids drive CsgA into an off-pathway by the formation of insoluble oligomers. In contrast, phloretin affected the solubility of CsgB, leading it in a soluble form and, therefore, compromising CsgA polymerization (Figure 1a) [98]. In a parallel work, it was reported that the flavonoids quercetin, myricetin, and scutellarein specifically inhibited biofilm formation of *S. aureus, S. hyicus*, *S. simiae*, and *S. saprophyticus*, which expressed Bap [99]. The flavonoids did not affect *bap* expression but reduced the formation of Bap amyloid-like aggregates (Figure 2c). Because the Bap N-terminal region adopts an unstable molten globule-like state, it was proposed that flavonoids bind this region, thus stabilizing the Bap native folded state and avoiding the polymerization of the amyloid aggregates. Interestingly, quercetin and myricetin, at least, were effective under in vivo conditions because they reduced *S. aureus* biofilm formation on catheters implanted in mice. Since BAPs are present in many pathogenic bacteria, the use of polyphenols to fight against biofilm associated infections could be widely extended [135].

## 4. Final Remarks

Numerous compounds with anti-amyloid properties have been identified to date. Some of them are effective at reducing biofilm formation in many bacteria. However, future directions of research on anti-biofilm molecules that target functional amyloids should include several aspects. First, it is necessary to consider that anti-amyloid compounds can have species-specific effects. They can inhibit biofilm formation of a certain bacteria but could have the opposite effect on the biofilm of other bacteria. This is the case for the plant flavonoids luteolin, myricetin, and quercetin, which inhibit the assembly of curli of *E. coli* and Bap amyloids of *S. aureus*, strongly reducing the extracellular biofilm matrix; in contrast, they enhance *P. aeruginosa* macrocolony biofilm formation and have no effect on *B. subtilis* biofilm formation [98]. In addition, EGCG was found to promote biofilm formation of *P. aeruginosa* and increase its resistance to tobramycin [136]. Under certain conditions, EGCG reduced the susceptibility to vancomycin, oxacillin, and ampicillin of *S. aureus* [137]. The cell envelope stress generated by some compounds could lead to an induced biofilm development and an increase in anti-microbial resistance. 

A second intriguing aspect is the cross-reactivity effect that the anti-amyloid molecules could have on human amyloids. Although many of the compounds able to inhibit bacterial functional amyloids also actively reduce human aberrant amyloids, it is conceivable that they could have a contrary effect. It is important to highlight that FN075 displays cross-reactivity with diverging activity. It inhibits CsgA fibril formation but increases the rate of α-synuclein fibrillation. This discovery reveals the need to assess cross-reactivity with several amyloidogenic human proteins when anti-amyloid molecules are designed [119]. 

Bacterial amyloids, such as curli, are major compounds of the extracellular matrix of gut microbiomes, which have been linked to autoimmune diseases, neurodegenerative diseases, and cancer [138]. However, bacterial amyloids of the gut may benefit the host by educating the immune system and reinforcing the epithelial barrier. This raises an intriguing question as whether the oral administration of anti-amyloid compounds would influence gut amyloids and therefore, have any influence on health and disease. The specific processes underlying the beneficial or detrimental effects of anti-amyloid drugs on gut amyloids require thorough analysis.

Finally, many of the compounds identified to date have been tested for their anti-amyloid and anti-biofilm effects using in vitro models. Biofilm formation is a multifactorial process in which many different molecular processes are involved. This means that not all of the components that are expressed in vitro will be produced in vivo. Therefore, future research should focus on confirming the anti-amyloid activity of the drug candidates using in vivo models. 

## Figures and Tables

**Table 1:** Classification of functional amyloids.

Amyloid Type	Locus	Amyloid Subunits	Bacteria	
Intrinsic amyloids				
Curli	*csgDEFG; csgBAC*	CsgA, CsgB	*E. coli, Salmonella*	[40]
Fap	*fapABCDEF*	FapC, FapB,	*P. aeruginosa*	[41]
MTP	*mtp*	MTP	*Mycobacterium tuberculosis*	[42]
Chaplins/Rodlins	*chpA, chpD, rdlA, rdlB; chpC, chpH; chpF, chpG; chpB, chpE*	ChpD-H, RdlB	*Streptomyces coelicolor*	[43,44]
Facultative amyloids				
PSMs	*psmα_1–4_; psmβ_1–2_; hld*	PSMα, PSMβ, δ-toxin	*S. aureus, CNS ^1^*	[17,45]
TasA	*tapA, sipW, tasA*	TasA	*B. subtilis, B. cereus*	[46,47]
Bap	*bap*	BapB-domain	*S. aureus, CNS ^1^*	[48]
Esp	*esp*	N-terminal domain	*E. faecalis*	[49]
P1	*p1*	AgII-C123 region	*S. mutans*	[50]
WapA	*wapA*	WapA	*S. mutans*	[51]
SMU_63C	*smu_63c*	SMU_63C	*S. mutans*	[51]

^1^ CNS: coagulase negative staphylococci.

**Table 2:** Anti-biofilm agents targeting bacterial amyloids.

Type	Amyloid Inhibitors	Anti-Biofilm Effect	Bacterial Amyloid Target	Eukaryotic Amyloid Target	Reference
Peptides	ANK6	*S.* Typhimurium	CsgA	Aβ	[87]
	DB3DB3	*S.* Typhimurium	CsgA	Aβ	[87]
	AP90	*S. aureus*	PSMα1	ND	[88]
	AFhPs	*S. mutans,* *S. sanguis,* *S. aureus,* *E. coli*	ND	ND	[89]
	P1	*S. mutans*	ND	ND	[89]
Proteins	TTR	*E. coli*	CsgA	Aβ, HepF-N	[89]
		*B. subtilis*	ND		
Antibodies	3H3	*S.* Typhimurium	Curli	Aβ, TTR, Tau	[90]
Molecular tweezers	CLR01	*S. aureus*	PSMα1	Aβ, α-syn, Tau	[91]
	CLR05	*S. aureus*	PSMα1	Aβ, α-syn, Tau	[91]
Curlicides	FN075	*E. coli*	Curli	Aβ	[92]
	BibC6	*E. coli*	Curli	ND	[92]
	VA028	*E. coli*	Curli	ND	[92]
Bioactive compounds	AA-861	*B. subtilis*	TasA	New1	[93]
		*S. mutans*	P1 WapA		[92]
	Parthenolide	*B. subtilis*	TasA	New1	[93]
Polyphenols	EGCG	*E. coli*	CsgA CsgB	Aβ, α-syn, Tau	[94]
		*Pseudomonas* sp.	FapC		[95,96]
		*S. mutans*	P1 WapA SMU_63c		[89]
		*S. aureus*	PSMα1PSMα4		[97]
	PGG	*Pseudomonas* sp.	FapC	Aβ	[95]
	Tannic acid	*S. mutans*	P1 WapA	Prion PrP, Aβ	[51]
	Luteolin	*E. coli*	CsgA	Aβ, α-syn	[98]
	Morin	*E. coli*	CsgA	Aβ, α-syn	[98]
	Myricetin	*E. coli,* *S. aureus*	CsgA Bap	Aβ, α-syn	[98,99]
	Quercetin	*E. coli*	CsgA Bap	Aβ, α-syn	[98,99]
	Phloretin	*E. coli*	CsgA CsgB	Aβ, αSA53T	[98]

ND: non determined; Aβ: Amyloid β; α-syn: α-synuclein; αSA53T: mutant form of α-synuclein.
